# The impact of COVID‐19 on aquaculture in China and recommended strategies for mitigating the impact

**DOI:** 10.1111/jwas.12886

**Published:** 2022-04-15

**Authors:** Yuan Yuan, Weimin Miao, Xinhua Yuan, Yunyun Dai, Yongming Yuan, Yunchong Gong

**Affiliations:** ^1^ Key Laboratory of Freshwater Fisheries and Germplasm Resources Utilization Ministry of Agriculture, Freshwater Fisheries Research Center, Chinese Academy of Fishery Sciences Wuxi China

**Keywords:** aquaculture, COVID‐19, Guangdong Province, Hubei Province, mitigation strategy and measures

## Abstract

We carried out a preliminary investigation to study the impact of COVID‐19 on aquaculture in China and identify the strategies and measures that have been taken by the Chinese Government. The investigation involved questionnaire surveys designed for all stakeholders along the industrial chain, including grow‐out farmers, seed producers, fish processors, fish traders, and feed companies engaged in the catfish sector in Hubei Province and the tilapia sector in Guangdong Province during the strict period of control and after these control measures were lifted. We also attempted to summarize the government interventions and measures taken by different stakeholders along the value chain to minimize the damage caused by COVID‐19 and support the recovery of different sectors in the aquaculture industry. We found that due to delayed harvesting, fish stocks were held‐up in ponds and normal farming was interrupted. Farmers and traders were more severely impacted by the pandemic than other sectors. Furthermore, a series of strategies and measures are recommended to cope with the pandemic and other similar risks in the future. We expect that this study will provide good evidence for international societies to support the aquaculture industry in minimizing the impact of the pandemic and the rapid recovery of the industry in the post‐pandemic period.

## 1 INTRODUCTION

The COVID‐19 pandemic is an ongoing coronavirus disease caused by severe acute respiratory syndrome coronavirus (SARS‐CoV‐2). The first infection was identified in December 2019 in Wuhan, China (Valencia, 2020). On January 23, 2020, the Wuhan Municipal Government took the decision to close all outbound pathways connecting the city with the outside,^1^ to temporarily shut down different types of commercial activities and public services and enforce strict lock‐down measures across the entire city of Wuhan. On January 24, a first‐class response mechanism for unexpected major public health emergencies was initiated in many provinces and cities across China.^2^ The responsibility of local governments was intensified to manage the crisis through the enforcement of relevant control measures, including the strict control of transportation and travel activities, canceling all forms of mass gathering events, and shutting down large business activities, in order to contain the spread of the epidemic. On April 8, the control measures relating to outbound travels from Wuhan City were lifted, all transportation returned to normal, and the social and economic activities gradually returned to normal gradually in China.^3^

As the largest aquaculture producer and largest player in the global trade of aquatic products in the world (FAO SOFIA, 2020a), aquaculture has become a mature food production sector with a strong market orientation in China. China's aquaculture is a sector with high work division and specialization, with strong linkages between upstream and downstream stakeholders (Gui, Tang, Li, Liu, & De Silva, 2018). Normal operation in this sector relies heavily on the industrial chain across all links. The COVID‐19 pandemic, and the measures enforced to contain the epidemic, have had a significant impact on food production systems (including aquaculture) and logistic systems (Minahal et al., 2020). The Chinese government has adopted various measures to effectively mitigate the immediate and short‐term effects of the epidemic on the aquaculture sector and has achieved good results. The long‐term impact of the COVID‐19 pandemic on the aquaculture sector will continue for a long period of time, particularly since the global pandemic has not yet been controlled effectively.

In order to comprehensively understand the direct impact of COVID‐19 on major stakeholders in China's aquaculture industrial chain, this study carried out an investigation on the impact of COVID‐19 on the Chinese aquaculture industry with selected cases, namely the tilapia sector in Guangdong Province and the channel catfish sector in Hubei Province. Tilapia and channel catfish are both important aquaculture commodities with significant importance for both domestic consumption and the international market (Yuan, Yuan, Dai, & Gong, 2017). Specifically, almost half of the farmed tilapia production in China is processed for export and approximately 30% of farmed channel catfish production is processed and destined for the foreign market (Dai, Yuan, Yuan, et al., 2019). Guangdong Province and Hubei Province are among the most important aquaculture areas in China, ranking first and fourth in terms of all aquaculture production in China in 2019 (BoF, 2020). Guangdong Province is the largest producer and exporter of farmed tilapia in China, in which the total production of farmed tilapia reached 740,000 metric tons in 2019. Hubei Province is the largest freshwater aquaculture province and one of the most important channel catfish farming areas in China; this is also the major production base for channel catfish seed for the whole country. The total production of farmed channel catfish in Hubei Province reached 40,000 metric tons in 2019 (BoF, 2020). Hubei Province was the focus of the first outbreak of the COVID‐19 epidemic, and Guangdong Province was among the provinces in China that were most hit by the epidemic. Both of these two farmed commodities rely heavily on external markets with well‐established industrial chain structures. The COVID‐19 pandemic, and the lock‐down measures enforced, were expected to have a more significant impact on the two subsectors.

Existing studies of COVID‐19 mainly focused on the impact of the pandemic on the food system or on small‐scale farmers in different countries such as Bangladesh (Islam, Khan, & Barman, 2021; Rafiquzzaman, 2020; Sunny et al., 2021), the Philippines (Manlosa, Hornidge, & Schlüter, 2021), Malaysia (Jomitol, Payne, Sakirun, & Bural, 2020; Waiho et al., 2020), the Mekong Region (Lebel et al., 2021), Scotland (Murray, Ives, Smith, & Moriarty, 2021), India (Mohanty, Mandal, & Thakur, 2020; Purkait, Karmakar, Chowdhury, Mali, & Sau, 2020) and Thailand (Chanrachkij et al., 2020). Most of these studies perceived problems related to the strict control of transportation systems, reduced demand for fish, and the decreased market price of fish. Other studies focused on policies to cope with the pandemic and sustainable recovery plans. Kiruba‐Sankar et al. (2021) recommended a policy framework to strengthen the planning and management of the freshwater aquaculture sector towards the path of sustainability. Jamwal and Phulia (2021) put forward the strategies for making different aspects of aquaculture and fisheries resilient to a future pandemic‐like situation. Zhang, Tang, Zhang, Sun, and Yang (2021) reported the government's pivotal role in stabilizing the supply chain and striking a balance between control requirements and efficiency required in trading activities. All these studies provided us with an overview and background of the impact of the pandemic around the world.

To identify the impact of the COVID‐19 pandemic on the farmed tilapia and channel fish sectors, we carried out an investigation of major stakeholders within the industrial chain, including foodfish farming, seed production, product processing, trades and markets, and feed manufacture. Our study provided good snapshots of the impact of COVID‐19 on the aquaculture industry in China by focusing on two specific cases. We also summarized the strategic and policy measures taken by the government at different levels and the sectoral players to alleviate the damage of the COVID‐19 pandemic on different sectors and to accelerate the recovery of the sector following the lifting of lock down measures across the country. We hope that our results can provide good reference guidelines for other countries to develop appropriate strategies and action plans to encourage adaptation and increase the resilience of the aquaculture sector to challenges such as COVID‐19, through transformative changes and innovation in farming systems, management practices, industrial chain structures and operations of the aquaculture sector.

## 2 MATERIALS AND METHODS

In order to understand the direct impact of the COVID‐19 pandemic on the major stakeholders in China's aquaculture industrial chain, we carried out an investigation of the impact of COVID‐19 on the Chinese aquaculture industry, focusing specifically on the farmed tilapia sector in Guangdong Province and the farmed channel catfish sector in Hubei Province. The survey was conducted from July to November 2020. A set of survey questionnaires were designed; each questionnaire targeted individual links in the industrial chain for farmed tilapia and channel catfish (Azra et al., 2021), including grow‐out producers, seed producers, processors, marketing/traders, and feed manufacturers. While focusing on the impact of the COVID‐19 pandemic on the operation and performance at different links along the sectoral chain, the questionnaires also covered the impact of the COVID‐19 epidemic on the household livelihood, daily life, and specific impacts on women engaged in the entire industrial chain (FAO, 2020b).

Based on the structure of the tilapia sector in Guangdong Province and the channel catfish sector in Hubei Province, survey samples for foodfish production, seed production, processing, marketers/traders, and feed manufacture units, were identified by survey coordinators in the two provinces. The questionnaire survey was carried out by the provincial survey teams in each province through face‐to‐face interviews and telephone interviews. In total, 45 questionnaires were collected and analyzed in each province. Data collection activities stopped when 45 responses were collected in each province, which allowed us to understand the general situation. Because the questionnaires were performed during the pandemic period, it was difficult to obtain a high number of questionnaires. Through the investigation, we found that the impact of the pandemic on the same sector was similar. The policy collection continued until we began writing the article. Through questionnaire analysis, we obtained response rates and we summarize them in the part of results.

The questionnaire targeting each stakeholder group within the industrial chain covered the following aspects:

- The profile of stakeholders: name, location, production scale/capacity, product species and types, production systems, and staffing
- Direct impact on the business operation and management of the surveyed farm/company during the epidemic (by the end of April 2020)
- Impact on the livelihood and daily life of households and women engaged in or associated with the industrial chain
- Forecasted lasting impact after the lifting of strict measures to contain the epidemic and to predict the operational performance in 2020 by the interviewed respondent
- The impact on external services used by stakeholders in the industrial chain
- The actions taken by the government to support the sector to mitigate the impact of the epidemic and the rapid recovery of the industry once the epidemic is well controlled, and the measures taken by the stakeholders to mitigate the impact of the pandemic
- Anticipated further support from the government to assist the aquaculture sector to recover in the post‐pandemic stage and build preparedness and resilience for facing future challenges such as pandemics and other hazards

Based on the survey results and mitigation measures taken by the government, enterprises, and farmers, this case study focused on the analysis of the specific mitigation and impact of the pandemic on the industrial chains for channel catfish and tilapia. We described the actual impact of the strict control measures on the channel catfish and tilapia industries in Hubei Province and Guangdong Province, the long‐term impact on the channel catfish and tilapia industries in China, as well as recovery after the lifting of strict control measures.

## 3 RESULTS

### 3.1 The impact of the COVID‐19 pandemic on different industrial chain links in the farmed channel catfish and farmed tilapia sectors

The lockdown and other related measures used to contain the COVID‐19 epidemic in the two provinces disrupted the normal operations of different links in the aquaculture industrial chain and had a significant impact on stakeholders, even though the lockdown and epidemic containment measures were different. Because the epidemic was under control and the major strict lockdown measures had been lifted by the end of March 2020,^4^ April 1 was used as the demarcation point for collecting data and information on strict epidemic control impact and post‐epidemic recovery performance (up to the end of the survey period at the end of November 2020). The survey also considered the expectations of different stakeholders in the industry chain with regards to the overall performance of their business in 2020. Analysis revealed differences in the impact of the pandemic on different links for both the channel catfish and tilapia industries.

- The impact of the pandemic on the farmed channel catfish sector in Hubei Province was more severe than the farmed tilapia sector in Guangdong Province. All channel catfish farmer respondents and 57% of tilapia farmer respondents reported that their normal feeding patterns were completely disrupted. The second major impact was the hold‐up in stock of market‐ready channel catfish and tilapia in the pond; this occurred in 90% of the surveyed channel catfish farms and 57% of surveyed tilapia farms. In addition, 85% of channel catfish farms postponed stocking or failed to start new production cycles. Other impacts included the interruption of basic services and an increase in transportation costs for production input in the early stages of the epidemic.
- Channel catfish farmers and traders were most severely affected by the COVID‐19 pandemic; the number of orders placed was reduced by 30–60% for channel catfish and 10–30% for tilapia compared with the same period last year. Both feed companies and processors were less affected.
- International trade of the farmed channel catfish and tilapia was more severely affected than the domestic market. New orders for processed channel catfish and tilapia were 60 and 15% lower than during the same period as the previous year, respectively, while the domestic sales of products were decreased by 35% for channel catfish and by 10% for tilapia.

The investigation also identified the specific impact of the pandemic on the production and operations of channel catfish and tilapia along different value chains. Several factors were involved, as detailed below.

**FIGURE 1:**
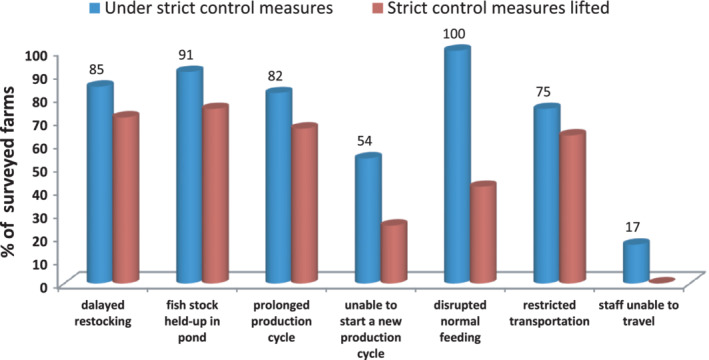
Impact on channel catfish grow‐out farming management and operation

**FIGURE 2:**
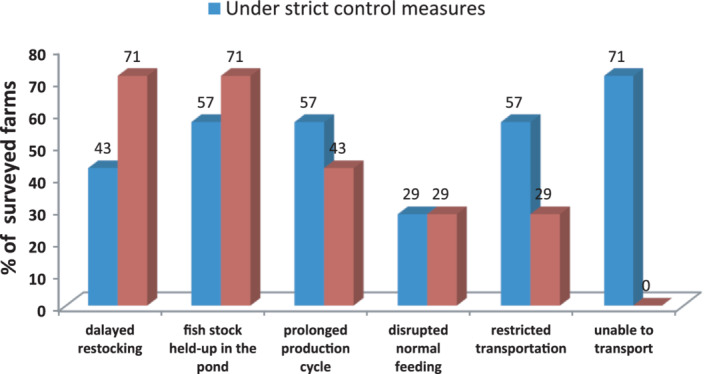
Impact on tilapia grow‐out farming management and operation

- Due to delayed harvesting, fish stocks were held up in ponds and the next farming cycle was prolonged (Figures 1 and 2).
- Due to certain epidemic containment measures, the normal management was interrupted, thus leading to poor fish growth (e.g., irregular feeding).
- Due to insufficient supply to the fish traders and the panic in international markets, international orders and the sales of two commodities were significantly reduced. For international trade, tilapia processors encountered breach/cancellation of orders/contracts by 20–50%. The surveyed tilapia processors indicated a decrease in new orders by at least 20% compared with the same period in the previous year.
- Due to increased production costs and delayed revenue, grow‐out farmers encountered financial difficulty in operation. Most respondents (>90%) forecasted that the production cost would be 10–15% higher than previous years due to increased feed cost, labor cost, and prolonged culture cycle.

We also investigated the impact on the livelihood of people along the value chain. We found that their income was reduced significantly due to fewer payments (e.g., the income of the catfish seed was reduced by more than 50%) and their families encountered financial difficulties (30–40% of the surveyed respondents). We also focused on the impact on women; this demonstrated that the burden associated with caring and educating children was increased and women were under additional pressure to keep the basic living conditions of their families (Chanrachkij et al., 2020).

The investigation indicated that the participants of the different sectors used various measures to mitigate the impact of the pandemic. These measures included prolonging the production cycle and reducing the potential loss of marketing, for example, by developing E‐commerce. The Chinese government also took strategies and interventions to support the basic living and normal farm operations during the lockdown period, such as unrestricted passage for food and key production material transportation, providing subsidies to the processing and trade companies so that they could purchase fish products, and providing financial assistance to households experiencing difficulties in managing basic living.

Based on the forecast of the surveyed respondents, the yield of farmed channel catfish and tilapia would be approximately 20% lower compared to the previous year; the production cost would increase by more than 10% and the profit would be 20–25% lower in 2020. The sale of farmed channel catfish and tilapia would be 20–30% lower in 2020 than the previous year. However, the prediction made by experts with regards to the production and sale of farmed channel catfish and tilapia at the whole country level was more optimistic; this was because of the anticipation of increased culture areas in the second half of the year to compensate for the loss of farmed fish production during the pandemic period.

### 3.2 Special impact on women associated with the channel catfish and tilapia industrial chain

Analysis showed that there was a much greater burden on women with regards to caring for and educating their children due to school closures. Women were also under greater pressure to maintain the basic living standards of their families. There was a significant impact on the time that women devoted to production management during the epidemic. More than 80% of the surveyed channel catfish grow‐out farmers reported that women devoted less time to production management. The survey results also showed that there was no significant difference between men and women engaged in the industrial chain links in terms of salary reduction and job layoffs. The epidemic affected more women engaged in channel catfish grow‐out farming than in tilapia grow‐out farming in terms of salary reduction and job layoff. Less than one‐third of the surveyed tilapia farmers indicated that the epidemic affected women/men in terms of salary reduction and job layoffs, while that of surveyed channel catfish farmers was 50%. In fish seed production, processing, and feed sectors, there was no difference in terms of salary reduction and job layoffs between female and male staff.

### 3.3 Actions for mitigating the impact of COVID‐19 on the aquaculture sector and governmental support for recovery

While taking firm actions to stop the spread of the epidemic, governments adopted strategies and measures to ensure the basic livelihood of the people involved and maintain operations in the industrial chain, such as relief aid to families with livelihood difficulties, guiding and supporting farmers and enterprises to maintain business activities, resuming production following the lifting of strict epidemic control measures, and minimizing the economic loss caused by the pandemic.

#### 3.3.1 The provision of emergency relief support to ensure the basic livelihood of stakeholders

During the strict epidemic control period, local governments provided unemployment insurance and minimum livelihood support to people engaged in the fish grow‐out, seed production, and fish processing sectors. For instance, the government provided nearly 10 million USD as living allowances for 141,900 vulnerable people^5^ in Jiayu County of Hubei Province.

In Guangdong Province, the government encouraged enterprises to avoid or minimize staff layoff by refunding 50% of the total unemployment insurance premium paid by the enterprise in the previous year. For families and individuals experiencing difficulties with their basic livelihood during the epidemic, certain measures were adopted in a timely manner, such as the provision of hardship subsidies and basic living materials. Since the outbreak began, the province had distributed a relief fund of 300 million USD.^6^ Almost two‐thirds of the surveyed grow‐out farms and half of the surveyed seed companies indicated that they benefited from the unemployment insurance or were included in the minimum livelihood security for urban residents. Half of the surveyed seed companies and fish traders, and two‐thirds of the feed manufacturers surveyed, received government hardship subsidies in shopping vouchers.

#### 3.3.2 Supporting the normal production and operations of all links in the industrial chain

In order to help fish farmers to maintain their production and management activities during the strict epidemic controls and resume production after the lifting of control measures, the Ministry of Agriculture and Rural Affairs of China (MARA) issued “The Guide of Technical Operation for Spring Aquaculture,”^7^ to guide seed production, grow‐out production, prevention and control of aquatic animal diseases, and the transportation of input supplies. Many Provincial Departments of Agriculture and Rural Affairs issued a “Notice on further improving aquaculture‐related work.” This notice aimed to guarantee the stable supply of aquatic commodities, ensure high‐quality aquaculture, and improve the dynamic monitoring of the market. Chinese fisheries extension agencies and research institutes also provided specific technical support and online guidance to farmers based on real‐time survey data and analyzed their impact. Also, government agencies strengthened the monitoring and early warning of aquatic animal diseases and constructed cold chain logistics facilities for storage and preservation.

For the fish trade sector, the government encouraged fish sellers to resume business operations with practical and feasible COVID‐19 prevention and control measures to ensure safety. The local government adopted green passage for the transportation of fresh and live food products and improved cold chain logistics systems. Measures were also taken to maintain the market circulation of aquatic products, improve product quality through branding, and organize various online promotion events as platforms to link producers and buyers. For example, the “National Aquatic Products Demand and Supply Information platform” (FAO, 2020c) was established to promote the production and marketing of aquatic products so as to solve the problems associated with “hard to sell” and “difficult to buy” fish products.

For the fish processing sector, the government published a special protocol on sanitation measures to prevent and control COVID‐19 while maintaining normal operations and production activities in the processing plants of aquatic products. To maintain the circulation of aquaculture products, the government supported the development of markets with automation and intelligent equipment. To facilitate fish exporters, government agencies accelerated the dissemination of information relating to trade control measures for aquatic products adopted in importing countries and implemented measures to facilitate the international trade of aquatic products. For instance, Fujian Provincial Marine and Fishery Bureau issued the “Health management measures for pandemic prevention and control in aquaculture and processing industry” guideline^8^ to make arrangements to prevent and control the pandemic in aquaculture and aquatic processing industries.

#### 3.3.3 Financial assistance

The government provided financial assistance to fish farmers and enterprises in the aquaculture industrial chain to cope with the difficulties caused by the pandemic, including tax exemption, government‐subsidized loans, special fund support, and the exemption of some government funds and administrative charges. Other measures adopted to support the sector included the exemption of fees for entry and exit inspection and quarantine, incentives for the purchase and storage of aquatic products, preferential loans (0.5% lower interest rate) and the extension of loan payback periods (up to 6 months).

To help enterprises to overcome financial difficulties during the epidemic, Guangdong Provincial government issued several financial assistance measures. For example, the government provided almost 20 million USD policy subsidies for processing and trade companies to purchase and store aquatic products and poultry during the epidemic. Such enterprises received a subsidy of 150 USD/ton when purchasing aquatic products. This strategy was targeted at processing and trade companies so that they could purchase and store a minimum of 43,690 tons of aquatic products.^9^ The government provided cash assistance of 150 USD/person for enterprise staff to return to work. The government also provided risk protection for enterprises that were affected by the pandemic, for example, the cancellation or reduction of orders.

**FIGURE 3:**
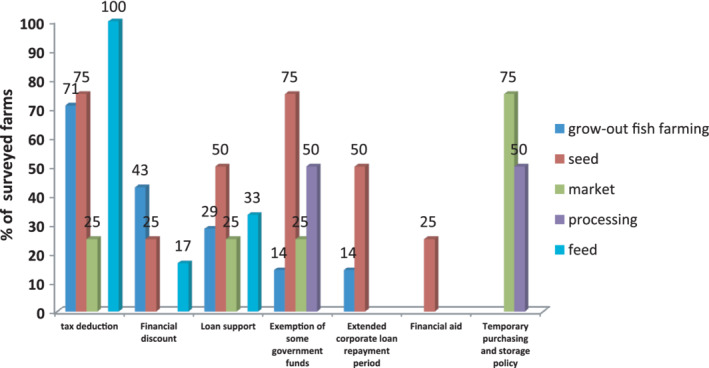
Surveyed farms and enterprises benefited from the financial assistance measures adopted by the Government during the epidemic period

The results of the survey relating to government financial assistance are summarized in Figure 3. These results indicated that the surveyed grow‐out farms, seed producers, fish traders, and feed manufacturers who benefited from tax exemption accounted for 71, 75, 25, and 100%, respectively. The surveyed grow‐out farms, seed producers, and feed manufacturers who benefited from government‐subsidized loans accounted for 43, 25, and 17%, respectively. The surveyed grow‐out farms, seed producers, fish traders, and feed companies who had priority access to national preferential loans accounted for 29, 50, 25, and 33%, respectively. In total, 14% of the surveyed grow‐out farms and 50% of surveyed seed producers were able to extend the payback period for outstanding loans. The surveyed grow‐out fish farms, seed producers, fish traders, and processing companies who benefited from the exemption of government administrative charges accounted for 14, 75, 25, and 50%, respectively. Three‐quarters of the surveyed fish traders, and half of the surveyed processing companies, benefited from the temporary policy subsidy for the purchase and storage of aquatic products.

### 3.4 Measures taken by sectoral stakeholders to mitigate the impact of the pandemic

The survey also attempted to capture measures adopted by the surveyed stakeholders to mitigate the impact of the pandemic on their business. The surveyed farmers indicated that their association/cooperative provided measures to assist them to address the impact of the epidemic, which included the adjustment of production plans, animal disease monitoring, and warning, technical guidance for business resumption, as well as measures to reduce economic loss by the jointly organized marketing of products, the sharing of sales channels, and the enrichment of product types. Over 80% of the surveyed grow‐out farmers indicated that they increased the proportion of processed product. One‐sixth of surveyed farmers adopted measures to increase product sales, such as creating new selling channels, new product types, prolonging the production cycle, and reducing feeding.

The mitigation measures taken by the surveyed seed producers included reduced stocking area (15%), lower stocking density (15%) and joining e‐commerce and online sales (5%). All respondents adopted mitigation measures, including the suspension of operations, reduced/suspended purchases, and selling at lower prices and larger quantities to address the immediate risk to operations.

The processing plants surveyed indicated that they adopted measures to mitigate the impact of the epidemic, including the improvement of processing techniques, producing higher‐value products, and increasing e‐commerce and online sales. To reduce the impact of the pandemic on the sale of fish products, the surveyed fish traders indicated that they increased the proportion of processed products and promoted the sales of their goods through live online broadcasts. The feed companies surveyed reported that they adopted various mitigation strategies to reduce the impact of the epidemic, such as enriching product types, improving the quality of feed, and promoting special packages to increase sales.

Under the stimulation of various policies, the operations of different aquaculture enterprises have returned to a normal production process, and all links within the industrial chain of aquaculture have been effectively returned to normal. Following the outbreak of the pandemic, the prices remained stable over the following period. The supply of aquatic products has remained stable and food security was guaranteed. Collectively, these data demonstrated the effectiveness of the response strategies.

## 4 RECOMMENDED GOVERNMENT STRATEGY AND MEASURES FOR MITIGATING THE IMPACT OF THE COVID‐19 PANDEMIC AND OTHER DISASTERS ON THE AQUACULTURE SECTOR

The COVID‐19 pandemic has had a significant impact on the aquaculture sector in China. In this study, we analyzed and evaluated the mitigation and relief measures during and after the pandemic. We found that the recommendations made to improve the resilience and minimize the impact on the aquaculture sector, as well as the livelihood of the stakeholders, could serve as useful reference guidelines for other countries to prepare for other pandemics and disasters.

### 4.1 Strategy

#### 4.1.1 Strengthen the early‐warning disaster system and our capability of risk mitigation

It is important to strengthen the early‐warning disaster system for public health emergencies and other hazards, and to retain capability in risk management, such as support monitoring and the in‐depth analysis of global aquaculture production and market changes (both international and domestic) during and after the pandemic. It is also important to provide strategic guidance to the sectoral stakeholders when facing similar challenges in the future. Moreover, it is important that we collect and disseminate timely information related to the import restrictions imposed by importing countries, provide technical support to enterprises engaged in the export of aquaculture production to meet the customs inspection and quarantine standards recognized by importing countries to improve the preparedness of the sectoral stakeholders in production planning, input procurement, and management adjustment.

#### 4.1.2 Promote innovation in aquaculture technology for better resilience and preparedness for disasters

It is important that we build the capacity of producers individually and collectively to apply different types of modern technologies to improve efficiency, such as distant and real‐time monitoring, and the manipulation of environmental culture conditions, auto‐feeding, and animal health management, such as vaccination and the development of disease‐resistant strains. Furthermore, the government should support infrastructure development to facilitate the adoption of the Internet of Things (this is an extended and expanded network based on the Internet, a huge network formed by combining various information sensing equipment within the network, realizing the interconnection of people, machines, and things at any time and any place), AI technology, and big data in aquaculture to increase the resilience and preparedness of the aquaculture sector to various risks such as COVID‐19 and other hazards in addition to the improved efficiency of production.

#### 4.1.3 Support the development of modern marketing mode for aquaculture products

The government should support the development of logistics systems and modern trade/marketing modes for aquaculture products, such as the E‐commerce platform, internet+, and the farm/retail chain‐to‐consumer direct sale‐delivery service mode (FAO, 2021). We should also increase resilience and adapt to the changing preferences of markets and consumers and develop new forms of products to adapt to the needs of modern trade/marketing modes, particularly when the conventional marketing mode is disrupted by external strikes. Furthermore, we should support enterprises to improve cold chain facilities for increased storage and preservation capacity for aquaculture products to meet the changing market requirement.

#### 4.1.4 Promote a dual circulation development pattern of the sector to satisfy the domestic and international markets with better resilience when facing disasters such as COVID‐19 and other market uncertainties

We should strengthen the analysis of international market demand and the patterns of changes when the markets are stricken by major human hazards and natural disasters and development‐specific strategy for the sector to tackle the challenges. We should also expand the demand of domestic markets for aquaculture commodities (Rafiquzzaman, 2020), which traditionally target the international market predominantly, and develop new forms of production to meet the changing preferences of domestic consumers.

#### 4.1.5 Improve social security and protection systems

The government should strengthen overall social security and disaster response systems and mechanisms through well‐coordinated financial and taxation policies, and insurance systems, at central and local levels to protect the entire sector when challenged by disasters such as COVID‐19 and other natural hazards. Furthermore, the government should strengthen the institutional arrangements and human capacity for providing emergency assistance and disaster relief to the sectoral stakeholders at different levels.

### 4.2 Specific measures for mitigating the impact of COVID‐19

#### 4.2.1 Provide emergency relief support to ensure the livelihood of stakeholders

It is important that we strengthen the security of essential livelihoods for the people who are severely impacted by the pandemic. During the pandemic, particularly the lockdown period, there was a significant impact on jobs and business operations, thus leading to reduced income and life difficulties in many households. Local government and public services should provide timely livelihood support to families who are in difficulty.^10^ It is also important to establish special assistance channels for families under low‐income social security and households encountering serious livelihood difficulties due to the COVID‐19 patient, or related epidemic control measures and provide timely emergency relief and medical assistance.

#### 4.2.2 Establish unrestricted logistics to ensure normal production and marketing in the agriculture sector

It is vital that we effectively implement green‐passage mechanisms to ensure the normal delivery of aquaculture inputs and avoid the build‐up of farmed stock (both food fish and seed). It is also important to support the farmers with regards to the timely harvesting and marketing of products and restocking to avoid significant disruption of the normal production cycle. Moreover, the operation of processing plants and the maximal purchase of raw materials from farmers, assisted by minimal protective prices, should be subsidized by the government.

#### 4.2.3 Support the normal operation of the sector through strengthened sectoral monitoring and analysis and information dissemination

It is vital that we strengthen our ability to monitor the operational status of processing plants, strengthen the input supply for farming operations and make changes in the consumer market and international trade to guide the normal operation of the entire value chain and strengthen the monitoring of farmed animal diseases (including the quarantine inspection of seed). We also need to monitor COVID‐19 infection in the production and marketing chain to avoid the transmission of COVID‐19 through farmed animals. We should also adopt sanitary measures, including the recording of personnel movement, health checking, and protective measures against COVID‐19 infection at farms and along the cold chain.

#### 4.2.4 Encourage processing plants and farmers to develop new forms of products that can adapt to new marketing modes and market needs

It is important that we support processing plants and farmers in developing new products, such as semi‐cooked, ready‐to‐eat, and prepared fish dishes, which are more suitable for online sales and distant delivery to replace live and fresh whole fish through conventional marketing and promote diversified and convenient fish products of high nutritional value and health standards as consumers pay more attention to health and food safety after the epidemic.

#### 4.2.5 Provide financial assistance to ease the difficulty experienced by the industrial chain

It is important that we implement preferential financial policy measures to companies and farmers along the aquaculture industrial chain for them to maintain normal operations and mitigate the impact of the pandemic, which include the extension of pay‐back periods for existing loans, subsidized bank interest, risk protection, the exemption of administration charges and delayed taxation. For example, it will be vital that we provide subsidies to companies for electricity, fuel, and logistics during the pandemic and to establish a fast track and simplify procedures for small and micro companies in the aquaculture industrial chain for obtaining funds from banks to overcome financial difficulties during the pandemic.

## 5 DISCUSSION AND CONCLUSIONS

The strict measures applied to contain the COVID‐19 epidemic have impacted the aquatic production. At the farm level (both grow‐out and seed production), road blockages, and restrictions on personal movement, have led to the discontinued supply of various production inputs, thus delaying stocking, interrupting normal feeding and management, prolonging the culture period and affecting the quality of the harvested products. Other specific problems caused by the COVID‐19 pandemic included weak market demand for farmed aquatic products, difficulty in selling fish, delayed harvesting (Kakoolaki et al., 2020), interrupted transportation of fresh and live fish products, high stocks of fish products at sale, and the loss of capital for some farmers and business operators; this last issue is consistent with that reported in a case study incorporating data from Bangladesh, Scotland and the U.S. (Hasan, Heal, Bashar, Bablee, & Haque, 2021; Murray et al., 2021; Van Senten, Engle, & Smith, 2021). In addition, farms and supporting companies are facing other difficulties in regaining full recovery of production, such as the lack of production capital, difficulty in hiring labor/staff, increased labor costs, and difficulty accessing loans and investment. One report revealed some positive impacts on the ecosystem and fish stocks (e.g., an increase in fish stock) due to the reduced disturbance of fishing activities (Islam et al., 2021).

To address the immediate impact of the COVID‐19 epidemic on the aquaculture sector, the Chinese central and provincial governments issued various guidelines and implemented relevant measures to guide local governments to support the normal operation of the aquaculture industrial chain. The important measures include an open green channel for the transportation of aquaculture products, the reduced build‐up of cultured fish stocks, increasing the purchase and processing of fish stocks held‐up in ponds to help farmers to maintain normal production cycles, supporting innovative forms of aquaculture products and marketing through E‐commerce platforms, and the provision of financial assistance to farmers, processing and marketing companies, such as loans with subsided interest and extension of the payback period for loans. These well‐targeted support measures have effectively maintained normal operations within the aquaculture industrial chain during the epidemic period. The effects of these measures can help to mitigate the impact of the pandemic and enable the speedy post‐pandemic recovery of the aquaculture sector following the easing of restriction measures to contain COVID‐19 in China. Data will help to identify the further assistance required by different stakeholders along the sectoral chain in terms of recovering from the disruption and restoring their normal production operations and daily life.

The COVID‐19 epidemic has been well‐controlled in China since April 2020 and the entire aquaculture sector industrial chain has gradually returned to normal operations, particularly in terms of production and the domestic market. However, the COVID‐19 pandemic could have a lasting impact on the purchasing behavior of consumers, market demand, and the prices of aquatic products. There is significant uncertainty with regards to overall aquaculture industrial performance in China, and globally, with the rebounding wave of the COVID‐19 pandemic after the summer of 2020 in many important economies. The Freshwater Fisheries Research Center research team will continue to monitor and analyze the full impact of COVID‐19 on aquaculture in China based on the eventual actual performance of the sector in 2020 and years to come.

## CONFLICT OF INTEREST

The authors declare no potential conflict of interest.

## AUTHOR CONTRIBUTIONS

**Yuan Yuan**: Investigation; data curation; formal analysis; writing the original draft. **Weimin Miao**: Conceptualization; methodology; validation; writing/reviewing/editing; supervision. **Xinhua Yuan**: Conceptualization; validation; writing/reviewing/editing. **Yunyun Dai**: Investigation; writing/reviewing/editing. **Yongming Yuan**: Investigation; writing/reviewing/editing; supervision. **Yunchong Gong**: Data curation; writing/reviewing/editing.

## Endnotes

^1^
Announcement of Wuhan Headquarters for Prevention and Control of Pneumonia Outbreak of Novel Coronavirus Infection (No. 1) [EB/OL]. January 23, 2020. http://www.wuhan.gov.cn/sy/whyw/202003/t20200316_960171.shtml.

^2^
The municipal government holds a work meeting on the prevention and control of pneumonia caused by new coronavirus infection [EB/OL]. January 25, 2020. http://www.shanghai.gov.cn/nw2/nw2314/nw2319/nw11494/nw44727/nw44734/nw44742/u21aw1423528.html.

^3^
Notice of Hubei Province New Coronavirus Infection Pneumonia Epidemic Prevention and Control Headquarters [EB/OL]. April 7, 2020. http://www.hubei.gov.cn/zhuanti/2020/gzxxgzbd/zxtb/202004/t20200407_2207131.shtml.

^4^
Notice of Hubei Province New Coronavirus Pneumonia Epidemic Prevention and Control Headquarters[EB/OL]. March 24, 2020, http://www.hubei.gov.cn/zhuanti/2020/gzxxgzbd/zxtb/202003/t20200324_2189256.shtml (in Chinese).

^5^
Over CNY 10 million distributed, ensure social relief and assistance in need under epidemic in Jiayu [EB/OL]. http://news.xnnews.com.cn/xwjj_1/202002/t20200228_1936518.shtml.

^6^
Guangdong Province has issued more than 2 billion CNY in aid since the outbreak [EB/OL]. March 24, 2020, https://life.dayoo.com/money/202003/24/154561_53223784.htm.

^7^
The Guide of Technical Operation for Spring Aquaculture[EB/OL]. February 12, 2020, http://www.gov.cn/xinwen/2020-02/12/content_5477673.htm.

^8^
Health management measures for pandemic prevention and control in aquaculture and processing industry[EB/OL]. February 14, 2020, http://hyyyj.fujian.gov.cn/xxgk/hydt/stdt/202002/t20200214_5196002.htm.

^9^
Guangdong allocated 120 million CNY to guarantee the “vegetable basket” for the purchase and storage of poultry, aquatic products, vegetables and fruits. [EB/OL]. March 11, 2020. http://www.gd.gov.cn/gdywdt/zwzt/yqfk/gdzxd/content/post_2928671.html.

^10^
Notice on Further Doing a Good Job in Protecting the People in Difficulties During the Epidemic Prevention and Control [EB/OL]. March 23, 2020. http://jda.cq.gov.cn/szfwj/55579.htm.
